# Evaluation of Local Rod and Cone Function in Stargardt Disease

**DOI:** 10.1167/iovs.63.3.6

**Published:** 2022-03-09

**Authors:** Krunoslav Stingl, Carel Hoyng, Melanie Kempf, Susanne Kohl, Ronja Jung, Giulia Righetti, Laura Kühlewein, Lisa Pohl, Friederike Kortüm, Carina Kelbsch, Barbara Wilhelm, Tobias Peters, Katarina Stingl, on behalf of the SORAPRAZAN consortium

**Affiliations:** 1 University Eye Hospital, Center for Ophthalmology, University of Tuebingen, Tuebingen, Germany; 2 Center for Rare Eye Diseases, University of Tuebingen, Tuebingen, Germany; 3 Department of Ophthalmology, Radboud University Medical Centre, 6500HB Nijmegen, the Netherlands; 4 Institute for Ophthalmic Research, Center for Ophthalmology, University of Tuebingen, Tuebingen, Germany; 5 Pupil research group, University of Tuebingen, Tuebingen, Germany; 6 STZ eyetrial at the Center for Ophthalmology, University Tuebingen, Tuebingen, Germany

**Keywords:** Stargardt disease, chromatic pupil campimetry, rod function, cone function

## Abstract

**Purpose:**

In this study, chromatic pupil campimetry (CPC) was used to map local functional degenerative changes of cones and rods in Stargardt disease (STGD1).

**Methods:**

19 patients (age 36 ± 8 years; 12 males) with genetically confirmed *ABCA4* mutations and a clinical diagnosis of STGD1 and 12 age-matched controls (age 37 ± 11 years; 2 males) underwent scotopic (rod-favoring) and photopic (cone-favoring) CPC. CPC evaluates the local retinal function in the central 30° visual field via analysis of the pupil constriction to local stimuli in a gaze-corrected manner.

**Results:**

Scotopic CPC revealed that the rod function of patients with STGD1 inside the 30° visual field was not impaired when compared with age-matched controls. However, a statistically significant faster pupil response onset time (∼ 40 ms) was observed in the measured area. Photopic CPC showed a significant reduction of the central cone function up to 6°, with a minor, non-significant reduction beyond this eccentricity. The time dynamic of the pupillary response in photopic CPC did not reveal differences between STGD1 and controls.

**Conclusions:**

The functional analysis of the macular region in STGD1 disease indicates reduced central cone function, corresponding to photoreceptor degeneration. In contrast, the rod function in the central area was not affected. Nevertheless, some alteration of the time dynamics in the rod system was observed indicating a complex effect of cone degeneration on the functional performance of the rod system. Our results should be considered when interpreting safety and efficacy in interventional trials of STGD1.

Stargardt disease (STGD1) is a rare autosomal recessively inherited macular dystrophy with central vision loss caused by biallelic mutations in the ATP-binding cassette transporter (*ABCA4*) gene.^1^ It is a retinoid flippase important for 11-cis-retinal and all-trans retinal recycling. Impairment of ABCA4 protein function leads to pathological and excessive accumulation of lipofuscin and bisretinoid A2E in the RPE. This process yields the activation of an apoptosis pathway and subsequently the death of RPE and photoreceptor cells.^2^ The *ABCA4*-associated retinopathy typically begins with central or pericentral vision loss and develops to various extents of macular atrophy. However, areas beyond the macula can also be affected and can lead to difficulties under dark adaptation, giving rise to fundus flavimaculatus, cone–rod dystrophy, and retinitis pigmentosa phenotypes.^3^^–^^6^

The recent review from Jeffery et al.^7^ summarizes findings on significant genetic variability and correlation between the clinical phenotype and the residual ABCA4 protein function. Biallelic *ABCA4* variants with an early childhood onset of central vision loss often develop into generalized cone-rod dystrophy.^8^^–^^10^ Further, it has been published that patients with the c.5882G>A;p.(Gly1961Glu) variant, the most prevalent disease causing allele, have a macular dystrophy with a characteristic bull's eye maculopathy or atrophy of the fovea typically surrounded by parafoveal flecks.^4^ In these patients, the functional outcome associated with a mild central vision loss often occurs with a later onset in the third decade of life.^4^ However, it has been shown that the phenotypic heterogeneity related to this variant can be caused by *cis*-acting genetic modifiers such as intronic mutations on the same allele.^11^ Thus, the current suggestion from Cremers et al.^12^ is to classify ABCA4 mutations to mild, intermediate, and severe or null *ABCA4* variants in connection with the associated distinct clinical phenotypes.

Morphological multimodal imaging of STGD1 performed with new modalities such as high-resolution optical coherence tomography or fundus autofluorescence imaging are extremely powerful tools, not only in the identification and general diagnosis of STGD1, but can also provide high precision to identify specific disease patterns associated with the genotype.^7^ In contrast with numerous morphological evaluations, the number of functional tests used in STGD1 is relatively small. Full-field ERG recordings are normal in many patients, indicating an isolated macular disease, but in some patients, especially in progressed cases, full-field ERG can show generalized retinal abnormalities with decreased photopic and scotopic responses. Reports from ERG recordings show only a limited correlation between morphological and functional changes,^13^^,^^14^ with no direct correlation between the clinical appearance, electrophysiological characteristics, and the specific mutation.^15^ Functional evaluations of the macular region in STGD1 conducted with microperimetry can provide a reliable measure of the macular sensitivity.^15^^,^^16^ Even more specifically, the study from Strauss et al.^18^ demonstrated a decrease in rod sensitivity in the macula for STDG1. Although currently it is possible also to perform a separate scotopic examination of rod sensitivity, the question on the separation of rod and cone function from the same local retina source is not trivial in a perimetrical, threshold type of measurement. To date, there is no approved treatment for STGD1. Patients benefit from auxiliary support, such as reading aids, sun protection, and expert consultations, but a causal therapy is not available. Standard adeno-associated virus vector–based gene supplementation therapy approaches are not achievable owing to the large size of the *ABCA4* transcript. Several pharmacotherapeutic clinical trials are currently underway, aiming to prevent or decrease the A2E accumulation. Additionally, dual vector–based gene therapy, or antisense oligonucleotide therapy aiming at correcting for splicing mutations and stem cell research, as well as CRISPR/Cas9-based therapies, are in preclinical testing.^19^^–^^21^

Owing to the large heterogeneity in the clinical phenotype of STGD1, but also in the progression speed, it is not easy to define an ideal morphological or functional readout to measure therapeutic effects. In this article, we introduce a novel functional test, namely, chromatic pupil campimetry (CPC), specifically designed to evaluate photoreceptor function at the local retinal level, targeting either the cone or the rod population.^22^^–^^26^ CPC measures the relative change in pupil size after local monochromatic stimuli and was presented in several recent publications describing normative values and expected range with testing specificity, reliability and reproducibility.^23^^,^^25^ In the functional evaluation of AMD, it demonstrated a high sensitivity to changes in disease state,^26^ and its application in patients receiving gene therapy for retinitis pigmentosa showed that CPC is a tool for an individual evaluation of the functional rescue of both cones and rods separately.^24^ In this study, we aim to evaluate the sensitivity of CPC in STGD1 by detecting areas and extent of photoreceptor dysfunction. Ultimately, data from this study can be further used as an objective functional end point in the evaluation of developing treatments for STGD1.

## Methods and Participants

### Participants

Examinations were obtained from 19 patients (aged 36 ± 8 years; 12 males and 7 females) with genetically confirmed *ABCA4* mutations, and a clinical diagnosis of STDG1 at baseline visit for an interventional trial (EudraCT no.: 2018-001496-20). The genetic report before the inclusion to the trial had to include at least two causal variants of the *ABCA4* gene and, in most patients, was based on panel sequencing or genome sequencing in a diagnostic–genetic setup. Visual acuity between 0.2 and 0.8 (decimal) in both eyes was one of the inclusion criteria. All participants underwent a full ophthalmological examination to exclude any possible additional interfering pathology, including best-corrected visual acuity testing, slit-lamp examination, fundus ophthalmoscopy, optical coherence tomography, and fundus autofluorescence imaging. Additionally, fixation stability was evaluated using mesopic microperimetry (MAIA; CenterVue, Padova, Italy). CPC was measured only in one eye with the other eye patched. Detailed characteristics of the subjects including genetic finding and preferred retinal locus (PRL) position distance to fovea calculated from mesopic microperimetry are presented in Table 1.

**Table 1.** Characteristics of Subjects With STGD1 Included in Our Study

				*ABCA4*	*ABCA4*	*ABCA4*	*ABCA4*	Fixation	
ID	Eye	Age	BCVA	Variant 1	Variant 2	Variant 3	Variant 4	Stability	Fixation
001	Right	44	0.2	c.1622C>T^*^	c.3113T>C^*^	c.5882G>A		Relatively unstable	Parafoveal (>3°)
002	Right	31	0.625	c.5603A>T	c.1622C>T^*^	c.2588G>C	c.3113T>C^*^	Stable	Foveal
003	Right	25	0.5	c.634C>T	c.5882G>A			Unstable	Foveal
004	Right	33	0.5	c.1622C>T	c.3113T>C	c.2588G>C		Stable	Foveal
005	Right	42	0.5	c.3322C>T	c.5714+5G>A			Stable	Foveal
006	Left	38	0.25	c.1622C>T^*^	c.3113T>C^*^	c.5882G>A		Relatively unstable	Parafoveal (<3°)
007	Left	34	0.2	c.3261A>C	c.5882G>A			Relatively unstable	Parafoveal (>3°)
008	Right	34	0.5	c.2588G>C	c.4234C>T			Stable	Foveal
010	Right	38	0.625	c.108delT	c.1912C>T			Stable	Foveal
011	Left	58	0.625	c.70C>T	c.5714+5G>A			Relatively unstable	Foveal
014	Left	38	0.625	c.1086T>A	c.5603A>T	c.6089G>A		Stable	Foveal
015	Right	35	0.625	c.5714+5G>A	c.5714+5G>A			Stable	Foveal
017	Right	31	0.8	c.2894A>G	c.5714+5G>A			Stable	Foveal
018	Right	36	0.5	c.5461-10T>C	c.6466C>T			Stable	Foveal
019	Right	46	0.625	c.5714+5G>A	c.5714+5G>A			Stable	Foveal
020	Right	42	0.4	c.5882G>A	c.6006-623_6549del			Unstable	Foveal
021	Left	18	0.25	c.1553A>C	c.5714+5G>A			Relatively unstable	Parafoveal (<3°)
023	Right	30	0.625	c.768G>T	c.2588G>C			Stable	Foveal
026	Right	34	0.5	c.1622C>T^*^	c.3113T>C*	c.5292C>T		Relatively unstable	Foveal

The fixation stability and fixation parameters are exported from mesopic microperimetry. BCVA, best-corrected visual acuity; ID, subject number.

* Typically c.1622C>T and c.3113T>C are inherited in *cis* as a complex allele.

In 14 subjects, the right eye was tested and in 5 subjects the left eye was the study eye. Thus, a direct averaging was not recommended, because the temporal and nasal retinal locations would be confounded. To ensure that no effect was driven by left to right eye retinotopic differences, in the case that the left eye was measured, all results were mirrored.

Twelve age-matched adults with normal vision served as controls (2 men, 10 women; aged 37 ± 11 years). The data of the control group have partially been published.^25^

All patients gave written informed consent before participation. The pupils were not dilated medically before the CPC measurement and none of the patients had reported taking any psychosomatic drug that could have influenced the pupillary response. The study was conducted in concordance with the Declaration of Helsinki and approved by the local Ethics Committee.

### CPC

For an objective evaluation of local rod and cone function within the central 30° visual field, separate CPC protocols for scotopic (rod-favoring protocol with blue stimuli) and photopic (cone-favoring protocol with red stimuli) evaluation were used. The stimuli were presented on a wide screen organic light-emitting diode monitor within the central 30° (LG organic light-emitting diode 55C7V). A correction of the refractory error is not used in our setup. A gaze-tracking algorithm was used to ensure correct retinotopy. Pupillary response and gaze tracking were recorded with an infrared camera with a sampling frequency of 100 Hz. Recording is started with 5 minutes of specific light exposure (weak blue light of the same intensity as stimulus used for the scotopic protocol). The photopic measurement after light adaptation consists of 41 red stimulation points inside of the 30° visual field. For the photopic protocol, red stimuli were presented on a dim blue background (baseline period 500 ms, stimulus radius 3°, stimulus duration 1 second, stimulus intensity 60 cd/m^2^, stimulus wavelength 620 ± 30 nm full width at half maximum, 1.7 × 10^−5^ watt, with an interstimulus interval of 4.5 seconds). An automatic control controls the return of the pupil to the baseline value, and, if this is not achieved, then the stimulus is repeated. The repetition of stimuli is also triggered if the gaze correction could not be performed (loss of gaze tracking or to large deviation from the fixation point). Beside these two conditions, also the eye blink artifact during stimuli presentation will trigger the repetition of a stimulation. The recording time of the photopic recording is therefore not fixed, but dynamic and subject dependent and approximately 8 to 9 minutes.

After the photopic measurement, the subject is dark adapted for 20 minutes. After that, the scotopic measurement is performed; dim blue stimuli were presented on a totally black background (stimulus radius 5°; stimulus duration 100 ms; stimulus intensity 0.01 cd/m^2^; stimulus wavelength 460 ± 30 nm full width at half maximum, 2.1 × 10^−8^ watt). Duration of scotopic measurement is in average approximately 6 to 7 minutes. Stimulus characteristics and protocols have been described in more details previously, including validation and the test–retest reliability profile.^23^^–^^25^

The CPC setup is currently only operational at University Hospital Tuebingen; all recordings reported herein were performed only in the Tuebingen site of the multicentric trial (EudraCT no.: 2018-001496-20).

### Data Analysis and Statistics

Pupil responses were normalized to the baseline pupil diameter and these relative values of amplitude were used for further analyses in accordance with the standards in pupillography.^27^ The map of the relative maximal constriction amplitudes (relMCAs) of the whole 30° area and the averaged relMCAs at specific eccentricities (0°, 6°, 12°, 20°, and 30°) were analyzed. The photopic protocol standard grid included additional four test points at 3° eccentricity (not performed in the scotopic protocol owing to a larger stimulus size of 5° compared with 3° for the photopic protocol). Therefore, pupillary responses from those stimuli at 3° eccentricity from the fovea and those from the fovea itself at 0° were averaged and compared with the foveal 0° responses of the scotopic protocol. Furthermore, the steepness angle of the line connecting relMCAs at peripheral locations in comparison with central locations was additionally used to estimate the severity of functional loss in the macular region. Mathematically, the steepness angle was calculated using average values from the same eccentricity and applying a linear fit to this value.

The time dynamics of the neuronal network controlling pupillary reflexes were evaluated through the analysis of the time from stimulus onset to pupil constriction onset (latency). This latency was calculated from the intersection between the estimated linear fit of pupillary constriction (period of 200 ms with constant first derivation) and the linear fit of the baseline period (0–500 ms).

To test for a group difference between patients with STGD1 and age-matched control subjects, two-tailed *t*-tests were used. The analysis of specificity and sensitivity of CPC in detecting STGD1 induced macular change was performed for the steepness angle with the receiver operating characteristic (ROC) curve.

Results are presented as mean ± standard deviation. A *P* of less than 0.05 was considered statistically significant. For eccentricity-specific analyses, the *P* value was adjusted according to Bonferroni correction 0.01 (5 nonindependent *t*-tests). All analyses were performed in MatLab (The MathWorks, Inc.; Natick, MA).

## Results

### Maximal Constriction Amplitude (relMCA)

**Figure 1.**
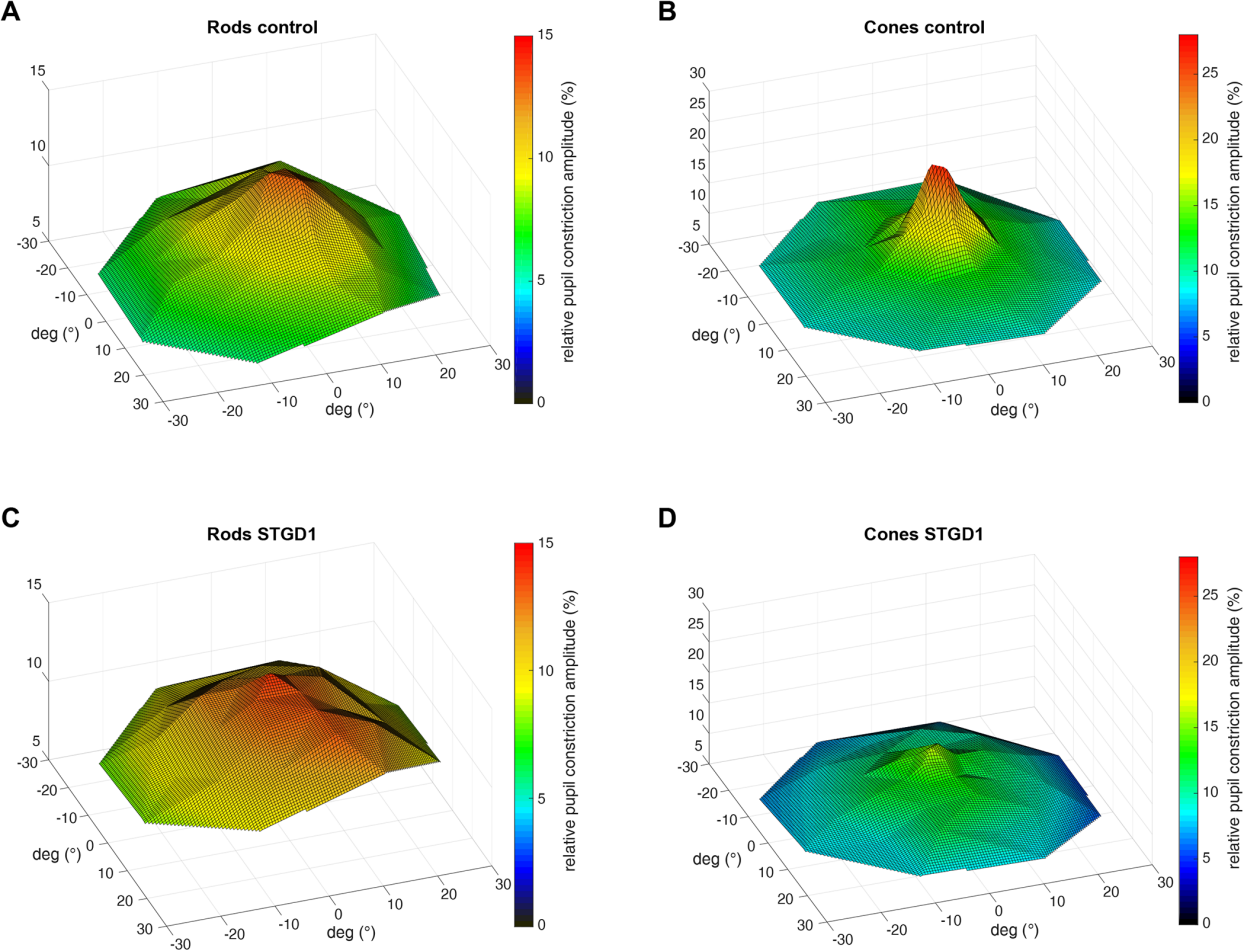
Topology of the CPC sensitivity maps (relative pupil constriction amplitude expressed as percent of the pre-stimulus baseline) for control group (*upper panel*, **A** and **B**) and STGD1 (*lower panel*, **C** and **D**) in the evaluated 30° visual field area. Responses (averaged values from all subjects) for scotopic stimuli (rods) are shown on the left and responses for photopic stimuli (cones) on the right.

The baseline pupil size did not show a statistical difference between groups in both conditions: Controls had an average baseline pupil (scotopic 6.0 ± 0.7 mm; photopic 5.4 ± 1.1 mm) comparable with STGD1 (scotopic 6.2 ± 0.9 mm; photopic 5.1 ± 0.8 mm). The retinal sensitivity maps for photopic and scotopic measurements averaged over the control group and STDG1 are presented in Figure 1 and reveal decreased relMCAs in STDG1 for the photopic protocol (mean relMCA fovea STGD = 13 ± 5%; mean relMCA fovea controls = 25 ± 5%).

**Figure 2.**
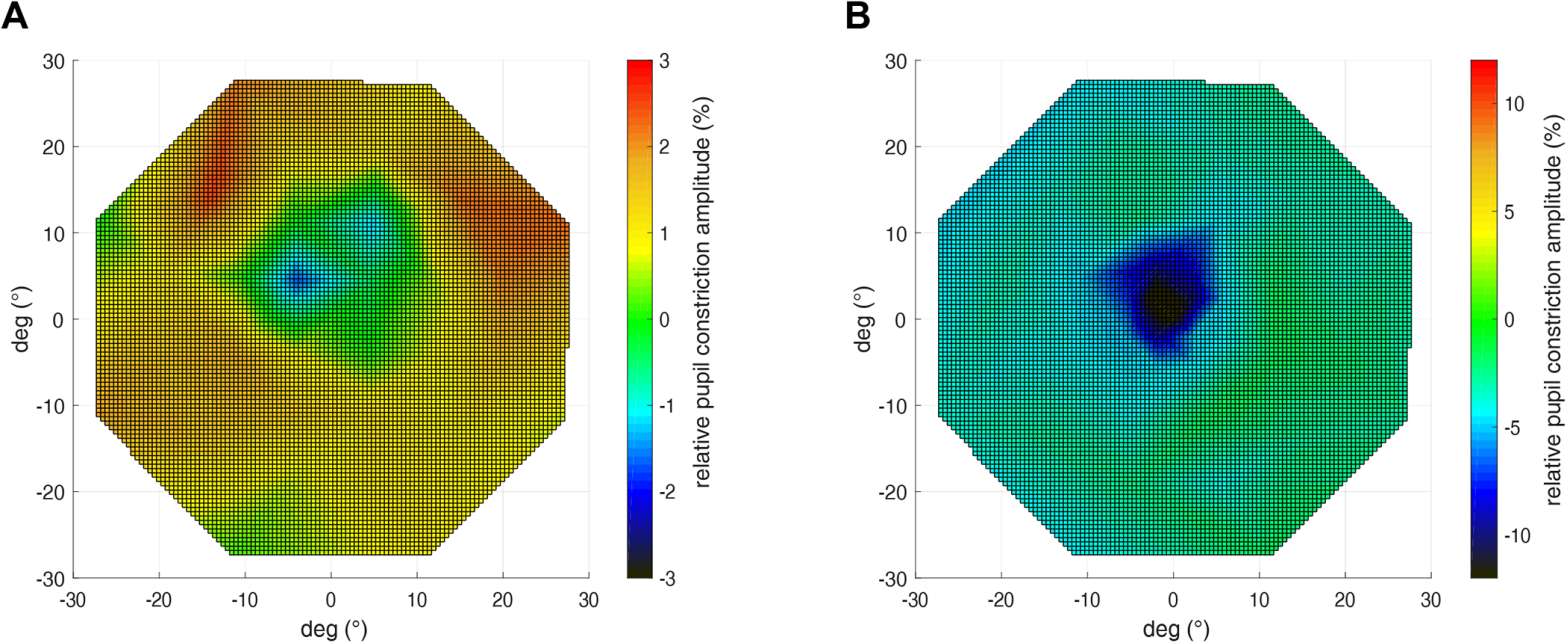
Differences of the CPC sensitivity maps as shown in Fig. 1 between the group averages (STGD1 – controls) in scotopic measurement **(A)** and photopic measurement **(B)**. The differences in relMCAs are color-coded: green = no difference; yellow-orange-red = stronger pupil responses (relMCAs) for STGD1, blue-black = reduced pupil responses (relMCAs) for STGD1.

**Figure 3.**
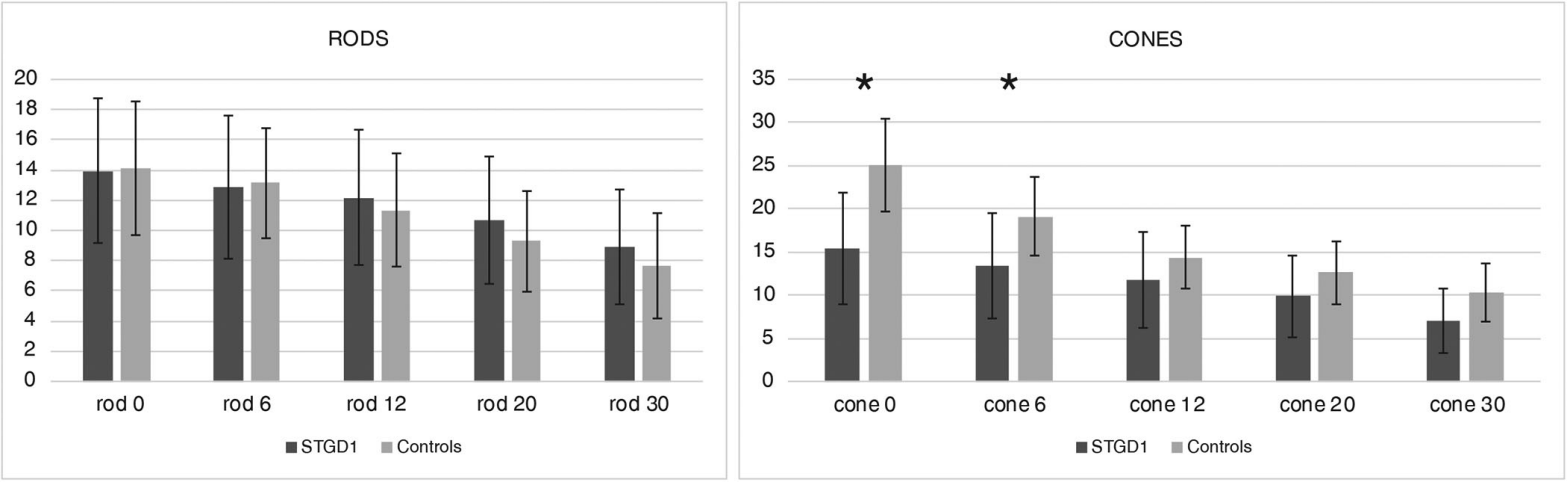
Amplitudes of pupillary responses (relMCA) for different eccentricities in scotopic (rod, *left*) and photopic (cone, *right*) measurements. In patients with STGD1, photopic pupil responses in the fovea (0°) and at 6° eccentricity were significantly smaller (*P* < 0.01) than in controls.

The difference between group averages (STGD1 vs. controls) for photopic and scotopic measurements are presented in Figure 2. We found similar responses in the macular region for scotopic responses with a small, non-significant reduction in the center and slightly increased rod responses in the periphery for STGD1. Pupil responses to photopic stimuli were significantly decreased in the center (*P* < 0.001; control relMCA = 25 ± 5%; STGD1 relMCA = 13 ± 5%) and at 6° eccentricity (*P* < 0.01 control relMCA = 19.0 ± 4.5%; STGD1 relMCA = 11 ± 5%), but did not differ between the two groups beyond the central 10° (statistics see Fig. 3 and the following).

### ROC Analysis for the Steepness Angle for relMCAs Between Peripheral and Central Photopic Responses as an Estimate for Macular Dysfunction

**Figure 4.**
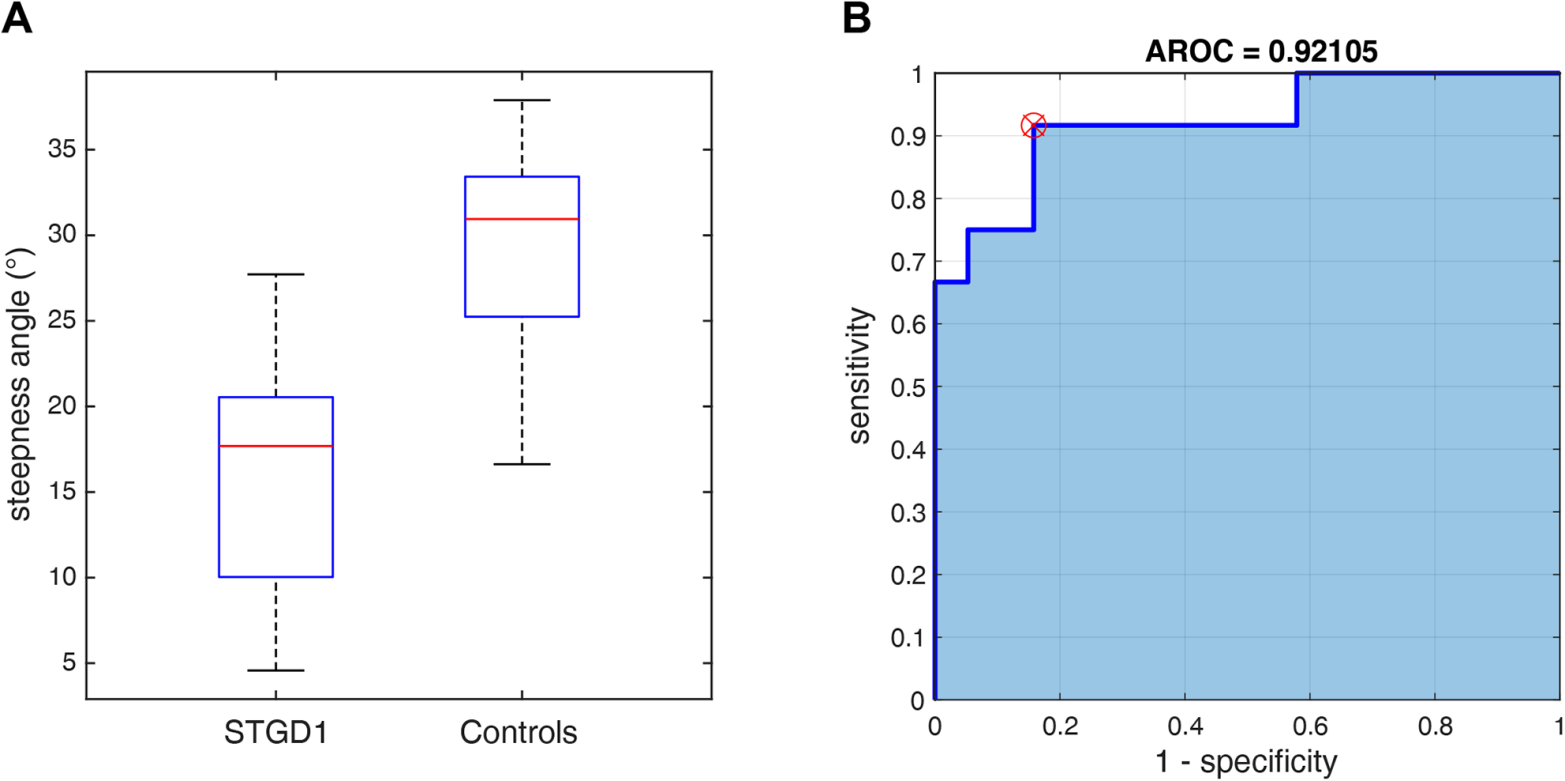
**(** **A)** Box plot of the steepness angle for controls and STGD1 group. **(B)** ROC analysis of steepness angle between control and STGD1 group (photopic condition).

A ROC analysis was performed to test the specificity and sensitivity of the steepness angle in detection of macular defects under photopic conditions in the patients with STGD1 compared with the controls. The results indicate that this factor has a high classification (area under the curve of 0.92) in detecting macular defects for the STGD1 group (Fig. 4).

### Time Dynamics – Latency to Constriction Onset

**Figure 5.**
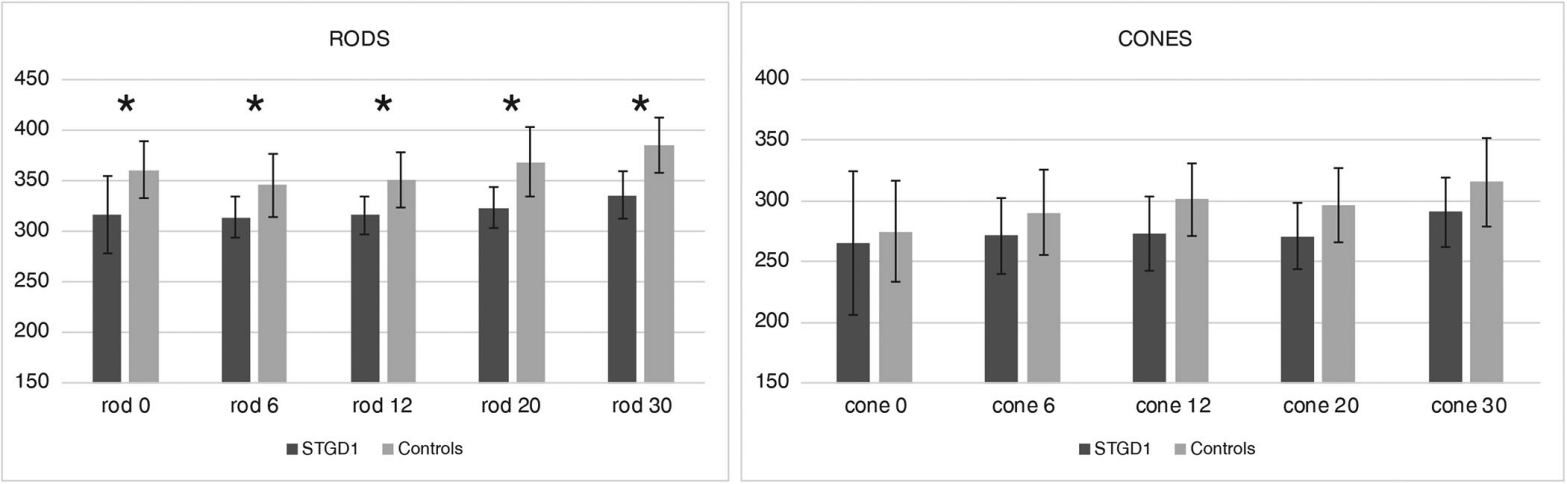
Temporal dynamic of pupillary responses for different eccentricities in the scotopic (rod, *left*) and photopic (cone, *right*) measurements. The latency to constriction onset in the scotopic measurements was significantly faster (*P* < 0.005) in the STDG1 group compared with controls at all stimulus locations.

Bar plots of the latency to constriction onset of the pupillary response for different eccentricities are shown in Figure 5. The onset of the pupil constriction was significantly faster (*P* < 0.005) in all eccentricities of the STDG1 group for the scotopic protocol compared with the control group.

### Correlation of Pupil Responses With Age

**Figure 6.**
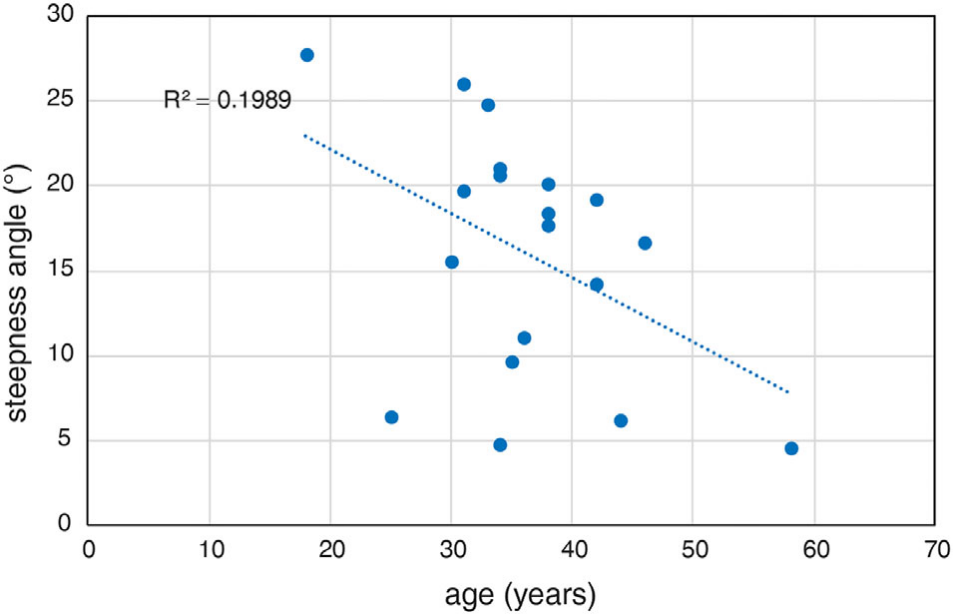
The correlation of steepness angle between the photopic measurement in STGD1 subject and age shows a statistical trend (*P* = 0.056).

The dependency of the steepness angle between relMCAs to central and peripheral stimulation in the photopic measurement and age for STGD1 subjects showed a trend not reaching statistical significance (*P* = .056). All other examination parameters including best-corrected visual acuity, relMCA, or the latency of the pupil responses at different eccentricities were far from statistical significance (Fig. 6).

## Discussion

These results validate the macular defect of cone function in early and middle stages of STGD1 with a novel objective functional diagnostic, namely, the CPC. This macular defect measured by the photopic CPC is characterized by a remarkable loss of the typical central CPC response hill of the relMCAs otherwise seen in the control subjects. The ROC analysis with the steepness angle of CPC response between peripheral and central stimulation under photopic condition resulted in an area under the curve of 0.92, indicating a good classification of the macular defect in patients with STGD1 in comparison with the control group. Although this deficiency in cone response is obvious for all patients, that functional defect is not absolute. All patients with STGD1 still have elicitable pupillary responses in the central region, indicating a substantial population of functioning cones. Our previous results pointed to a connection between the pupillary response to monochromatic local stimuli and the density of responding photoreceptors cells in the respective stimulated region.^23^^–^^25^ Thus, the mean decrease of photopic CPC results in patients with STGD1 for approximately 40% in the fovea or 20% at 6° of eccentricity could be interpreted as a decrease in cone density for approximately 40% in the fovea region, and a 20% decrease in the cone density at 6° of eccentricity. We found hints that this presumed density decrease of cones is present beyond the central macular region, but the difference in parafoveal retinal areas did not reach statistical significance. The approximation of a 40% cone decrease in the central region is in good agreement with earlier measurement conducted with adaptive optics imaging.^28^ The authors in that adaptive optics imaging study concluded that patients with STGD1 show a disturbance in cone density of up to approximately 4° to 5° eccentricity with a 50% decrease in cone numbers at the fovea region in comparison with the control group.

The progression of the disease expressed as a decrease of the steepness angle showed a possible correlation with patients’ age. Although this correlation presents only a trend, interestingly, no other readout such as the averaged relMCA, time onset of pupillary responses, or best-corrected visual acuity showed a similar relationship with age. The lack of correlation of these readouts is likely related to the genetic heterogeneity within our cohort, which presents a limitation in establishing better predictors for functional degeneration. The evaluation of the time dynamics of the pupil response shows that the cones in the whole tested area, but also in the area of damage, are properly connected to the inner retina. This finding may indicate that the degeneration does not exceed photoreceptors. In contrary to this finding, our earlier study in patients with AMD showed a prolongation of the foveal pupillary response latencies of approximately 80 ms.^26^ This change in time dynamics in patients with AMD is probably caused by a degeneration cascade from photoreceptors to the inner retina, which does not seem to be the case in STGD1. Although there are substantial age differences between the reported AMD and probable disease severity and the STGD1 cohort, the age-matched control groups of both cohorts had comparable results. Therefore, aging itself is not the main factor in the prolongation of the pupil response onset.^26^

In contrast with the clearly visible defect in the cone function in STGD1, we did not observe this effect in the rod system. The higher susceptibility of cones to STGD1 are known.^28^^–^^31^ The absence of a functional rod defect in these patients could be driven by their genotypes^30^ or by the fact that only subjects in early and middle stages of STGD1 have been enrolled in this trial. The sensitivity maps of rods even indicate some level of increased responses in comparison to the control group in the regions beyond 20°. This finding is in contrast with previously published data regarding the sensitivity of rods in the macula for STGD1.^18^ The explanation for this mismatch can be simple. The CPC is a measurement in which relatively strong scotopic stimuli are used and readouts are predominantly driven by the number of responding photoreceptors. In previous publications, the threshold type of perimetrical measurement was used. This measurement depends on the sensitivity of rods to detect weak stimuli. Therefore, although rods do lose their sensitivity, they are still sensitive enough to react to CPC stimuli and drive the pupil reaction. Therefore, the comparison of CPC data from STDG1 and the control group indicates that there was no substantial loss of rod photoreceptors in our cohort of patients with early and mild STGD1. Moreover, a potential lack of cone inhibitory function should be taken into account. This can be seen also for instance in subjects with *CNGA3*-achromatopsia, in whom a hyperactivity in the rod system has been shown by full-field pupillography.^32^ Very interesting findings are also seen in the time dynamics of the rods. In subjects with STGD1, the rod response time from the central macula measured by onset of pupillary responses was significantly shorter for approximately 40 ms than in the control group. To our knowledge, this study is the first time that this kind of change has been reported. A fast central cone degeneration could lead to a possible rod rewiring as described in the animal models of achromatopsia. Haverkamp et al.^33^ showed that, in those models, rods can reconnect to optic nerve cone–specific pathways. Alternatively, a lack of cone function can change the retinal light adaptation status and influence the gating mechanisms and information processing speed in the retina. Thus, data from our study indicate some fundamental changes of signal transfer from the outer to the inner retina; however, the exact mechanisms of these changes need to be investigated. Even if these mechanisms are beyond the scope of the data presented here, the clinical implication of this complex interaction must be considered. Any therapy aiming to restore photoreceptors in STGD1 could be faced with potentially conflicting results. For instance, a successful restoration of cone function could lead to improved inhibition of the rod system and, therefore, the reduction of response and increase in the response time. In the same time, however, any damage to the rod system during a therapeutic intervention would result in the same observed behavior. Therefore, in clinical trials for STGD1, we suggest a multimodal approach in testing rod function,^23^ and a careful interpretation of results in the situation where these complex interactions between rod and cone system are to be expected.

A limiting factor of this study is the fact that patients with STGD1 do not always have the PRL in the fovea; thus, the retinotopic stimulation could have been altered by this shift. The analysis of PRL in our cohort from microperimetric examinations showed that only in two subjects (Table 1), this would result in a substantial misplacement of stimuli from the CPC retinal maps. In other subjects, the size of the stimuli is large enough to ensure a proper stimulation according to morphological maps. The statistical significance did not change for any of factors even if these two subjects with PRL far from fovea region are removed from analysis.

In conclusion, the CPC measurement revealed several new characteristics of the STGD1-related degeneration of photoreceptors. First, the functional degeneration in early and middle phases of STGD1 is predominantly affecting cones. The loss of cones is highest in the foveal region, but is not absolute and does not seem to affect the interconnection with the inner retina. Second, changes in the rods’ time dynamics in affected regions indicate some level retinal network alteration up to the mid periphery, which should be considered in future therapeutic interventions. In the current setup, CPC presents a strong complement to classical retinal functional testing. With its previously shown high reliability and repeatability, this objective evaluation of the local rod and cone function respectively may be a valuable modality for testing the retinal function in therapeutic interventions for STGD1.
